# Advancements in surface-enhanced femtosecond stimulated Raman spectroscopy: exploring factors influencing detectability and shapes of spectra

**DOI:** 10.1515/nanoph-2024-0272

**Published:** 2024-12-06

**Authors:** Patryk Pyrcz, Sylwester Gawinkowski

**Affiliations:** Institute of Physical Chemistry, Polish Academy of Sciences, 01-224 Warsaw, Kasprzaka 44/52, Poland

**Keywords:** FSRS, SE-FSRS, SERS, coherent Raman scattering, plasmonic antennas

## Abstract

A combination of femtosecond stimulated Raman scattering and surface-enhanced Raman scattering, termed surface-enhanced stimulated Raman scattering (SE-FSRS), was proposed to leverage both temporal precision and sensitivity for advanced molecular dynamics analysis. During the initial successful implementations of this approach, unexpected spectral distortions were observed, and several potential explanations were proposed. Further progress in this novel technique and its broader implementation requires a profound understanding of the factors influencing the shape of the registered spectra and the underlying mechanisms. Here we present findings on how pulse energy and excitation wavelengths affect SE-FSRS spectra, emphasizing the influence of a strong broadband background on spectral dispersion. These insights contribute to understanding the complex mechanisms underlying SE-FSRS and suggest methods to improve the control and application of this spectroscopic technique, highlighting its potential to provide deeper insights into molecular dynamics. This work represents a significant step toward exploiting SE-FSRS for advanced analytical applications.

## 1 Introduction

Femtosecond stimulated Raman scattering (FSRS), introduced approximately three decades ago, has emerged as a pivotal innovation in the domain of nonlinear spectroscopy [1], [2]. Distinguished by its ability to capture vibrational structural dynamics with temporal resolution equalling or surpassing the oscillatory period of nuclear motions, FSRS has swiftly evolved, offering profound insights into reaction dynamics [3], [4], [5], [6]. Its unparalleled ability to investigate a diverse range of systems, with many of which being inaccessible through alternative methodologies, has established it as an essential instrument for molecular behaviour analysis. The foremost advantages of FSRS over the inherently weak spontaneous Raman process include the generation of stronger, more directional signals due to constructive interference from individual scatterers and the capacity for time-resolved observation of vibrational modes. This temporal coherence significantly enhances the detected signal intensity, potentially outperforming incoherent Raman by orders of magnitude.

In parallel, surface-enhanced Raman scattering (SERS) has revolutionised vibrational spectroscopy by enabling the detection of molecules at substantially lower concentrations [7], [8]. This advancement is mainly attributed to the excitation of plasmon resonances in metallic nanostructures, which enables them to function as optical antennas and focus light down to the nanoscale. Consequently, the optical response of molecules adsorbed on metal surfaces is significantly amplified, making Raman spectroscopy feasible for the detection of trace molecules and single-molecule vibrational analysis [9], [10], [11]. The characteristics of plasmon resonances (e.g. amplitude, position, and linewidth) can be modified by adjusting the nanostructures’ size and shape, achieved through self-assembly or nanolithography, each with unique benefits, limitations, and a propensity for introducing imperfections [12].

Considering the potential of both FSRS and SERS, their combination can further boost signal strength from molecular ensembles, promising quicker detection speeds for Raman-based chemical sensors and enhancing overall signal acquisition efficiency. Noteworthy advancements in surface-enhanced femtosecond stimulated Raman scattering (SE-FSRS) spectroscopy, especially its potential in single-molecule-level dynamics studies [13], underscore its potential for detailed observation and manipulation of quantum oscillators under standard conditions. Developing single-molecule SE-FSRS into a reliable technique demands an in-depth exploration of the intricate physics within plasmonic nanocavities. Critical areas of focus include understanding molecular-temporal coherence, heating effects on metal electrons and lattice, thermally induced molecular damage under pulsed light, and the third-order response of plasmonic antennas without the molecule present, laying the groundwork for significant progress in this vibrant area of research.

Progress in refining SE-FSRS control has been incremental to date. The team led by Van Duyne has notably advanced this domain by examining how sample thickness and the concentration of colloidal solutions of gold nanoantennas affect the SE-FSRS signal-to-noise (S/N) ratio and signal intensity [14]. The impact of excitation wavelength has been identified as a significant factor affecting the intensity and shape of bands in the spectra [15]. Furthermore, their work on assessing the impact of laser repetition rates, specifically comparing 100 kHz and 1 MHz FSRS systems, has provided critical insights [16]. Additional research has explored the application of 80 MHz lasers on dry samples, revealing that nanoparticles on glass substrates significantly enhance detection sensitivity, enabling single-molecule sensitivity [13]. These approaches have uniformly resulted in SE-FSRS spectra with distinctive dispersive band shapes. In contrast, impulsive stimulated Raman spectroscopy (ISRS), which captures stimulated Raman scattering signals in the time domain, does not produce these dispersive shapes, highlighting a critical knowledge gap and control challenge that limits the further development and application of SE-FSRS.

**Figure 1:**
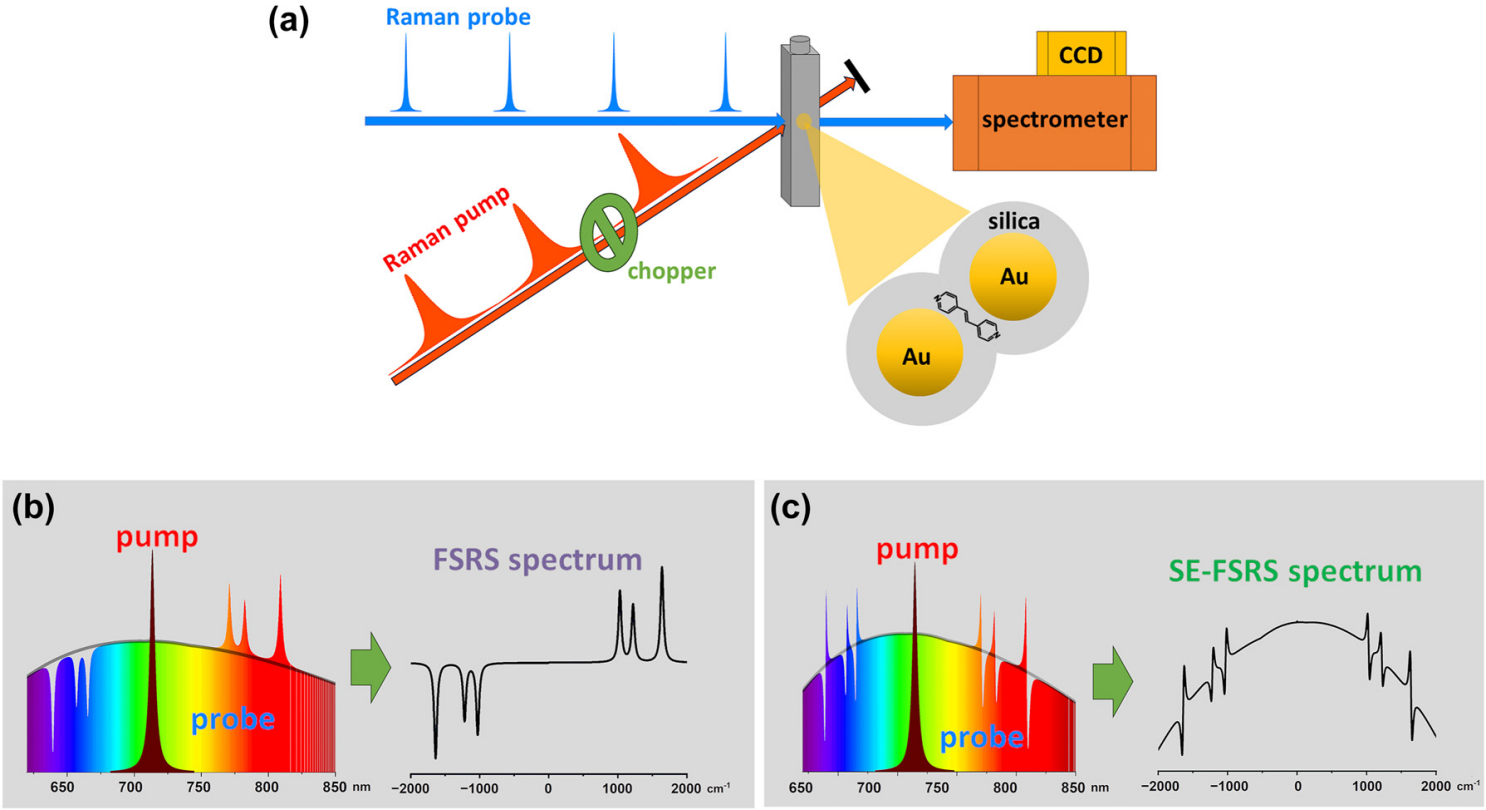
Schematic presentation of the optical excitation scheme in FSRS measurements (a) and the scheme of FSRS (b) and SE-FSRS (c) spectrum extraction from the measured spectral intensities distribution of the probe beam.

The appearance of dispersive Raman band shapes in SE-FSRS spectra (Figure 1), markedly different from the symmetric bands typically observed in standard FSRS experiments, and their variability across different experiments, suggested the presence of additional mechanisms in the SE-FSRS spectrum formation process compared to standard FSRS spectra. A likely factor responsible for this difference is the use of metal nanoparticles and their plasmonic properties. Based on the limited experimental data available for SE-FSRS, several physical mechanisms have been proposed to explain the strong Fano-type asymmetry observed in these spectra [17], [18], [19], [20]. However, the small number of experimental datasets, which exhibit significantly different dispersive shapes, has so far hindered the definitive identification of the underlying mechanism or the proposal of a new one that fully explains all observed effects. Identifying or developing such a mechanism that allows for theoretical predictions and complete consistency with experimental data is crucial for the further advancement of SE-FSRS spectroscopy and the exploitation of its immense potential.

To resolve this issue, a systematic investigation of the factors influencing SE-FSRS spectra is required, achieved by varying the relevant parameters. Our research delves into the factors affecting the dispersive characteristics of SE-FSRS spectra, including pulse fluence and a broad spectrum of excitation wavelengths. Additionally, we have found that SE-FSRS spectra are consistently influenced by a strong broadband background. The presence of this complex-shaped background signal, which may affect the dispersiveness of the bands, has not been previously reported. Furthermore, our examination of monodisperse gold bipyramid antennas has uncovered a relationship between the peak position of the background and the fluence of the plasmon resonance. Through this comprehensive analysis of factors influencing SE-FSRS spectra, we aim to shed light on the underlying mechanisms that define SE-FSRS spectral behaviour, thereby facilitating improved control and broader utilisation of this spectroscopic method.

## 2 Materials and methods

### 2.1 Nanoparticles preparation and characterisation

We conducted SE-FSRS measurements utilising two types of plasmonic nanoantennas: silica-coated spherical gold nanoparticles, each comprising an oligomer core (e.g. a dimer, trimer or tetramer) functionalised with embedded *trans*-1,2-*bis*(4-pyridyl)ethylene (BPE), and monodisperse silica-coated gold bipyramids.

Silica-coated, BPE-functionalized spherical gold nanoparticles (silica/BPE/AuNPs) were synthesised using methods previously described in the literature [21], [22], [23]. A typical experiment used 25 mL of AuNPs, with the concentration adjusted to achieve a maximum extinction of one at the plasmon resonance band. A 1 mM ethanolic solution of BPE was added to the AuNP colloid to allow for oligomer formation. The aggregation of monodisperse AuNPs was controlled in real time by measuring extinction spectra. Having obtained the appropriate amount of oligomers, 40 µL of a 1 µM aqueous solution of (3-aminopropyl)triethoxysilane (APTES) was added to the reaction mixture under constant stirring. Then, after 10 min, 400 µL of a 1.2 % aqueous solution of sodium silicate was added to the reaction mixture. The reaction mixture was left to stir continuously for 48 h. As a result, a thin layer of silica shell on AuNP oligomers was obtained. To obtain a thicker silica coating, the Stöber process was carried out, which included the hydrolysis of tetraethyl orthosilicate (TEOS) as a silica precursor [24]. The reaction mixture was concentrated to a volume of approximately 4 mL. Then, 27 mL of ethanol was added to 4 mL of this mixture. Subsequently, 2.32 mL of ammonia solution and 17.2 µL of TEOS were added to the reaction mixture under constant stirring. The reaction mixture was left for another 48 h. After this period, the mixture was centrifuged (15 min, 5,500 rpm) and washed with water three times to remove excess off chemicals.

Silica-coated gold bipyramids (silica/AuBPs) were produced according to methods described in the literature [25]. AuBPs (12 mL) were purified *via* two rounds of centrifugation at 7,000 rpm for 10 min. The precipitate was redispersed into water (12 mL), followed by subsequent addition and mixing of CTAB (0.1 M, 200 µL), TEOS (1.75 wt % in EtOH, 400 µL) and NaOH (0.1 M, 120 µL) under gentle shaking. The reaction was kept at room temperature under gentle shaking for 12 h, during which silica was deposited on the AuBPs to form the silica-coated AuBPs. The resultant samples were centrifuged at 5,000–6,500 rpm for 10 min. The precipitate was redispersed into water (6 mL) for further use.

For both types of plasmonic nanoantenna, morphological (SEM) and optical characterisation (UV/VIS/NIR extinction) were performed before and after FSRS experiments. A detailed description of synthesis protocols, reagents, materials, and characterisation is provided in the Supplementary Materials (SM).

### 2.2 Spontaneous Raman and SERS measurements

Raman and SERS spectra were recorded on a home-built microspectroscopy system described in detail previously [26]. Briefly, a beam from a laser diode (HL6545MG or HL6748MG, Thorlabs) was passed through a tuneable wavelength bandpass filter (VersaChrome TBP01-704, Semrock) and a tuneable optical density filter (NDC-50C-4M, Thorlabs). It was then directed to the microscope and focused on the sample. Measurements of solutions were performed using 10 mm quartz cuvettes with a 30-mm focal length objective and a laser power set to 1 mW. For measurements of dry SERS samples deposited on microscopic cover glass, an oil immersion objective (CFI Apochromat TIRF 100XC, NA = 1.49), Nikon objective was employed, and the laser power on the sample was maintained at 200 μW. The backscattered light from the sample was filtered by a long-pass filter (VersaChrome TLP01-704, Semrock) and analysed by a spectrograph (Kymera 328i, Andor) equipped with a CCD camera (iXon Ultra DU-888U3-CSO-EXF, Andor). SERS spectra of the colloids were recorded using a PMMA cuvette (Brand), whereas Raman spectra were recorded in a quartz cuvette (111-10-40, Hellma).

### 2.3 FSRS and SE-FSRS measurements

FSRS and SE-FSRS measurements were carried out using the home-built setup (Figure 1(a)), which was described in detail previously [27]. A Yb:KGW laser (Pharos, Light Conversion) generating 200 fs pulses at 1,030 nm with a repetition rate of 1 kHz was used to produce both the Raman pump beam and the Raman probe beam. The efficient spectral shift and compression of pulses from a femtosecond laser system produced picosecond Raman pump pulses, having a broadly tuneable narrow bandwidth (∼10–20 cm^−1^) [28]. A femtosecond probe (supercontinuum) was generated by focusing a small portion of the laser pulse onto a sapphire plate. The Raman pump and probe beams were crossed at a narrow angle and focused into a beam spot size of approximately 50 µm in the sample. The colloidal solutions of nanoparticles were flowed through a 1-mm quartz cuvette (137-1-40, Hellma) during the SE-FSRS measurements to avoid photodegradation. After passing through the sample, the probe beam was spectrally analysed using an Andor Shamrock SR 500i monochromator equipped with an Andor Newton U971N CCD camera.

## 3 Results and discussion

### 3.1 Effect of pulse fluence on SE-FSRS spectra

SE-FSRS spectra were recorded from colloidal solutions of BPE-functionalized AuNPs oligomers. This experiment marks the first successful recording using a 1 kHz excitation repetition rate system, demonstrating its compatibility with the most widely available FSRS equipment. Previously, SE-FSRS signals collected from aqueous colloidal solutions were recorded at frequencies of 100 kHz [14], [15], [29], [30], [31]. Previous experiments demonstrated that increasing the repetition rate of Raman pulses from 100 kHz to 1 MHz while maintaining the average power on the sample and, thus, reducing the fluence per pulse results in an improved S/N [16], which suggested that a significant decrease in the repetition rate could result in the signal being obscured by noise. Here, we show that SE-FSRS spectra may be acquired at 1 kHz by adequately controlling the pulse fluence, which is critical for the detectability of such spectra.

**Figure 2:**
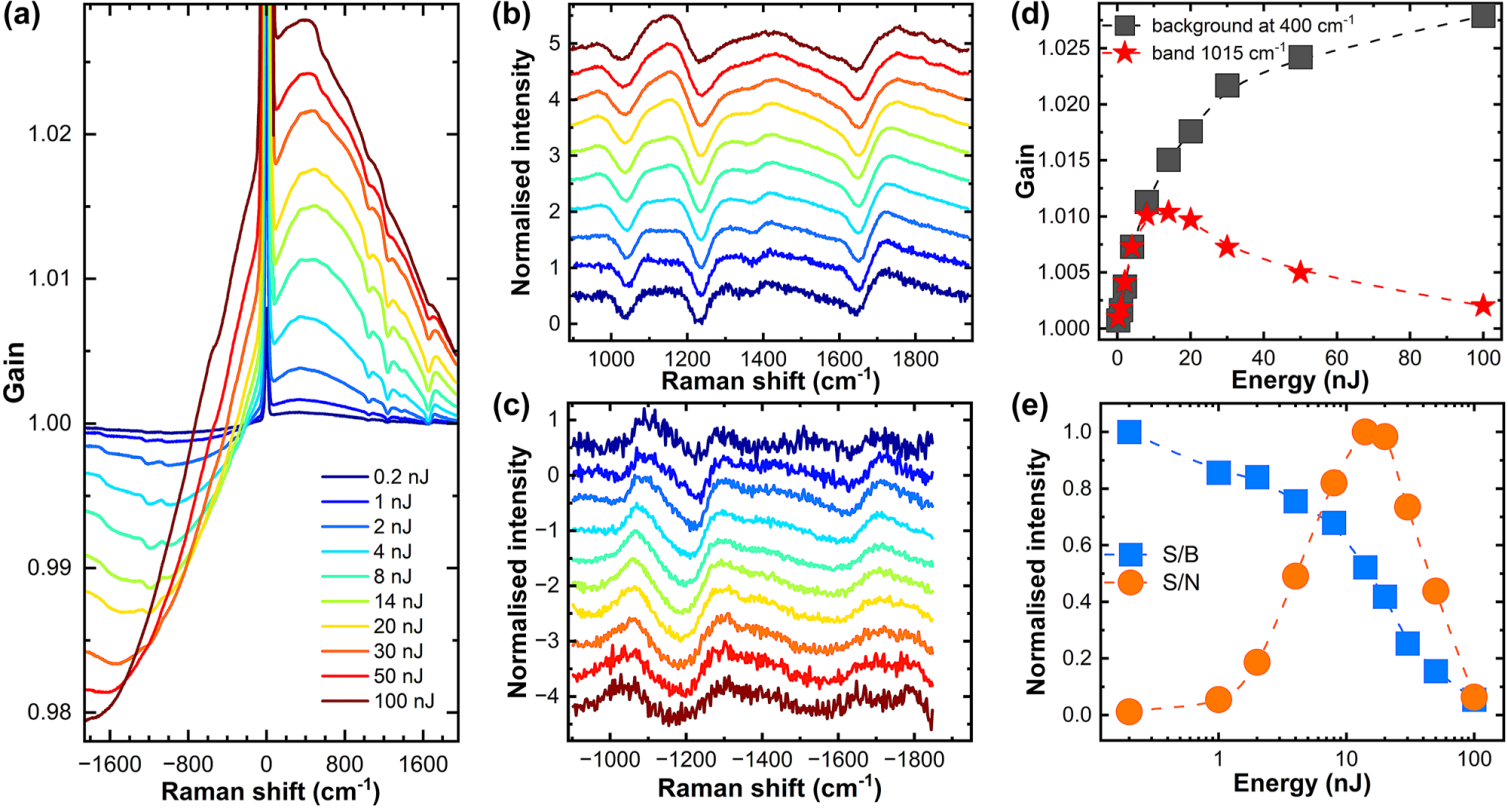
Pulse fluence dependence of SE-FSRS signal obtained for silica/BPE/AuNPs. SE-FSRS spectra registered for different Raman pump pulse energies (a). The baseline corrected and normalised Stokes (b) and anti-Stokes (c) parts of SE-FSRS spectra as a function of Raman pump pulse fluence. (d) Raman pump pulse fluence dependence of the 1,015 cm^−1^ BPE band amplitude (black squares) and broadband background amplitude at a 400 cm^−1^ Stokes shift registered at various Raman pump pulse fluence. (e) Raman pump pulse fluence dependence of the signal-to-noise (S/N) ratio for the 1,015 cm^−1^ BPE band amplitude (orange dots) and the signal-to-background (S/B) ratio registered at different Raman pump pulse fluence. Spectra are arbitrarily offset to enhance visibility in (b) and (c).

Figure 2 illustrates the dependence of the SE-FSRS spectra on the Raman pump pulse fluence. The acquisition time for a single spectrum was 5 min. With this acquisition time held constant, the fluence of a single Raman pump pulse was varied. Similar to observations in previous studies on comparable samples, the SE-FSRS spectra revealed a dispersive Fano-like band structure [29], [30]. The spectral positions of the bands were consistent with those observed in spontaneous SERS and with FSRS of BPE dissolved in DMSO (Figure S4 in Supplementary Materials).

Intriguingly, the SE-FSRS spectra exhibit a broadband background, appearing as a gain on the Stokes side and featuring a counterpart with negative amplitude on the anti-Stokes side (Figure 2(a)). As the Raman pump pulse fluence increases, the intensity of the background grows almost linearly for low excitation energies but begins to saturate at Raman pulse energies higher than 3.2 μJ/cm^2^ (Figure 2(e)). In addition, the amplitude of the dispersive Fano-like spectral features related to the Raman scattering of BPE increases linearly with the laser pulse fluence only up to approximately 3.2 μJ/cm^2^, but, unlike the background, saturates at approximately 5.6 μJ/cm^2^ and then starts to decrease linearly when passing 8.0 μJ/cm^2^ despite further increases in pump pulse fluence. This quenching-like behaviour may account for previous unsuccessful attempts to capture the SE-FSRS signal at a repetition rate of 1 kHz [32]. Increased Raman pump pulse energies also led to broadening of dispersive spectral features. The observed change as a function of pump pulse fluence aligns with the previously noted deceleration in the rate of increase in the SRS band amplitude with increased Raman pulse energies observed in ISRS experiments [33]. However, these experiments documented the effects only up to pulse energies of approximately 20 nJ, and a decrease in signal was not observed at higher pulse energies levels. At these pulse energies, the electric field at the focal point of the beam reaches approximately 10^8^ V/m. Due to plasmonic enhancement, we can assume that this field in the nanogap between nanoparticles may increase by approximately two orders of magnitude. This intensified field could be responsible for the photodecomposition of BPE molecules located in such a hotspot. Therefore, for pulse fluence above 3.2 μJ/cm^2^, the rate of this degradation process is so high that it cannot be compensated by the relatively slow exchange of the sample in the focal point, even with the forced flow of the colloid.

As can be seen in Figure 2(d), the increase in the fluence of the pumping pulse leads to a maximal S/N under a pumping pulse fluence of approximately 5.6 μJ/cm^2^; however, at the same time, the ratio of the amplitude of the Raman bands to the background intensity (signal to background, S/B) quickly decreases (Figure 2(e)). Additionally, at pulse fluence above 3.2 μJ/cm^2^, broadening of the Raman bands and distortions in the background can be observed (Figure 2(b) and (c)). Therefore, also considering the increased probability of photodecomposition of the sample at higher pulse energies, the optimal excitation fluence appears to be approximately 3 μJ/cm^2^.

The decrease and broadening of the SE-FSRS features’ amplitude at higher Raman pump pulse energies were almost fully reversible upon decreasing the pump pulse fluence. The only exception was that the total amplitude of the signals was slightly lower after previous measurements conducted with pulse fluence of 50 μJ/cm^2^ and above. This reduction in signal amplitude was attributed to a higher rate of irreversible photodecomposition of the sample under higher pump pulse energies, which occurred despite implementing a continuous flow of the colloidal solution through the excitation point using a flow cuvette.

Notably, when attempting to measure without the colloid flow, despite long acquisition times and the use of pulse fluence close to the optimum (3.2 µJ/cm^2^), the obtained spectra had a structure that was practically indistinguishable from the noise. Similarly, when the pulse fluence on the sample was in the range of 1 mJ/cm^2^, which is typically used for solutions of non-plasmonic samples, the signal of SE-FSRS bands was not visible. This observation suggests that successfully registering the SE-FSRS spectra requires choosing relatively small (approximately 2–5 μJ/cm^2^) Raman pump pulse energies and obligatory flow of the colloidal solution through the beam focus spot. Under these conditions, no change in the extinction spectrum of the sample was observed after a single 5-min measurement. However, illuminating the sample over multiple hours resulted in slight changes in the spectra, indicating very slow photodegradation of the sample but with a negligible effect on short-time measurements.

### 3.2 Effect of temporal overlap of Raman pump and probe pulses on SE-FSRS spectra

**Figure 3:**
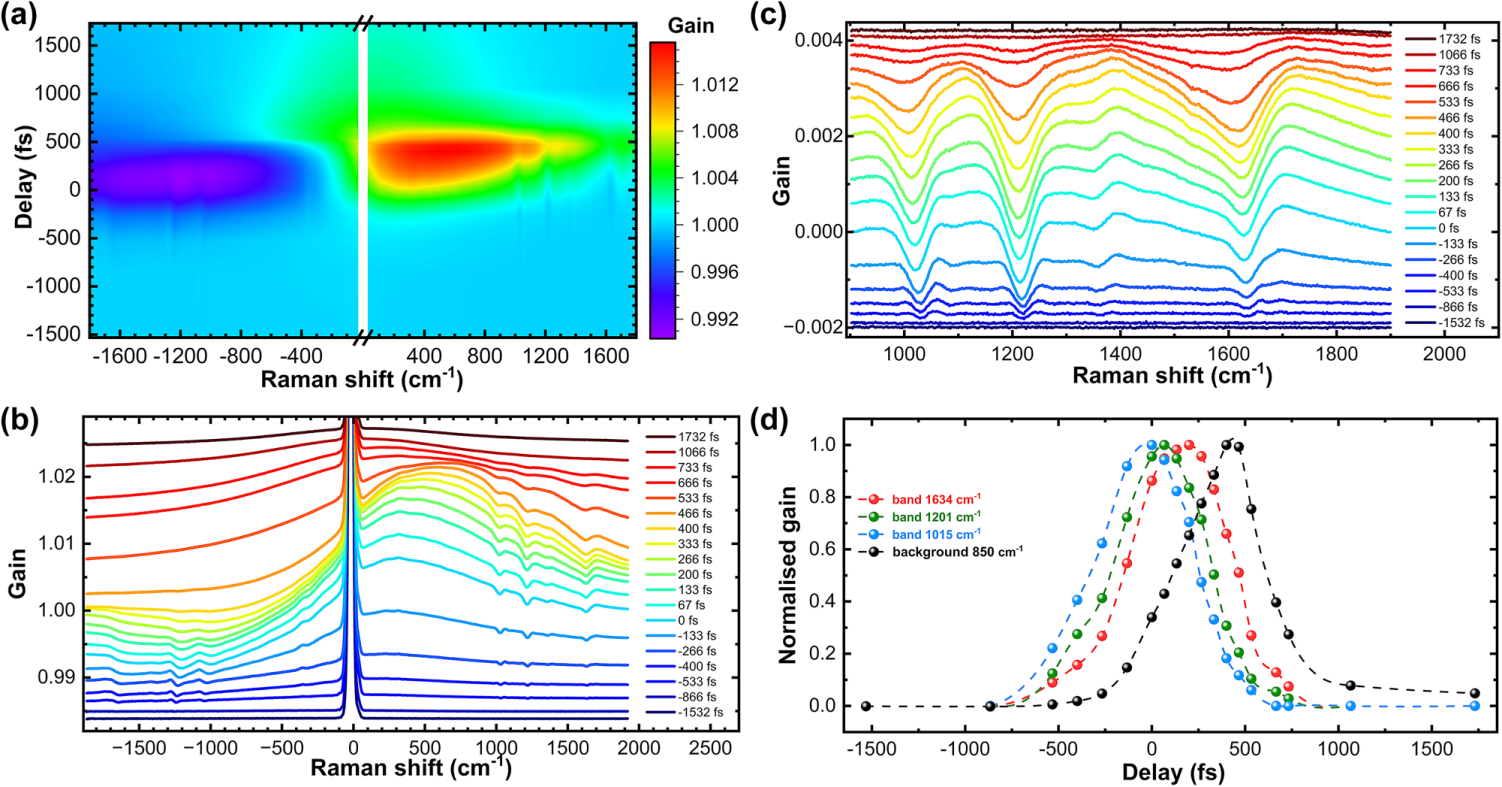
Time delay dependence of the SE-FSRS signal between the Raman pump and probe pulses for the silica/BPE/AuNPs (a) and (b). (c) Baseline-corrected Stokes-shifted parts of SE-FSRS spectra as a function of pump-probe time delay. (d) Gain dependence of Raman bands at Stokes shifts of 1,015, 1,201 and 1,634 cm^−1^, along with the background at 850 cm^−1^, as a function of pump-probe time delay. Spectra are arbitrarily offset to enhance visibility in (b) and (c).

The SE-FSRS spectra of silica-coated BPE-functionalized nanoantennas, obtained by varying the time delay between the Raman pump and probe pulses, are presented in Figure 3. The wavelength of the Raman pump beam was tuned to 665 nm to align it with the spectral range of the plasmon resonance band. Similar experiments were conducted to measure the FSRS signal of BPE dissolved in DMSO to draw comparisons with conditions lacking plasmonic resonance (Figure S5 in Supplementary Materials). The zero-time delay (∆*t* = 0 fs) was arbitrarily chosen as the point at which the intensity of the Raman bands was maximised. A negative time delay is defined when the probe pulse arrives before the peak of the Raman pump pulse.

The peak position of the Stokes component of the broadband background appeared to shift with the temporal delay between the Raman pump and probe pulses (Figure 3(b)). Initially, the peak was near the laser line, then gradually moved toward the red side until the band achieved its highest amplitude, and then it shifted back toward the laser line. This phenomenon is discussed further in Section 3.4.

A significant change in the dispersive shape of the Raman bands can be observed. The increase and then decrease in the amplitudes of these bands due to pump-probe time delay were accompanied by changes in their shape (Figure 3(c)). Initially, dispersive bands had a width of approximately 25–30 cm^−1^, similar to the FSRS spectra of the BPE solution (Figure S8, Supplementary Materials), as limited by the temporal and spectral characteristics of the equipment. With increasing delay, additional broadening was observed (similar to what was seen with increased pulse energies), resulting in a loss of spectral resolution. The observed changes in the shape of SE-FSRS spectral lines were qualitatively analogous to behaviours previously observed in FSRS measurements of compounds in solutions, particularly for those conducted with pre-resonant excitations [34], [35]. The changes in SE-FSRS band behaviour can be explained by drawing an analogy to those changes observed in FSRS spectra as a function of pump-probe time delay, considering the truncation of the vibrational dephasing time [30]. When the femtosecond probe pulse occurs after the picosecond pump pulse reaches its temporal maximum, the detected vibrational coherence is truncated early, resulting in broader vibrational peaks. Conversely, when the probe pulse coincides with the earlier part of the pump pulse duration, the peaks appear narrower due to the complete sampling of the vibrational coherence time. The peak intensity reaches its maximum when the pulses overlap at their temporal maxima, where coherence generation is most efficient. Our findings are in contrast with those presented by Frontiera et al. [30], who reported no observable changes in the nanoantenna line shapes with variations in the pump-probe time delay.

We observed that the position of the broadband background shifted with the pump-probe time delay (Figure 3(b)). The appearance of the background and its subsequent temporal evolution were time-delayed relative to the Raman bands of BPE (Figure 3(d)). The maximum gain of the background appeared approximately 400 fs after the peak of the 1,634 cm^−1^ band of BPE. The temporal width of the Raman bands roughly corresponded to the duration of the pump pulse. However, the temporal profile of the background evolution showed an additional longer component that did not decay to zero and persisted longer than the duration of the pump pulse.

The above-mentioned results indicate that the beginning part of the fluence in the pump pulse is sufficient to initiate the process responsible for generating the observed background. Given that the temporal profile of the probing pulse does not exceed 200 fs, the underlying process responsible for background generation must be significantly faster. In this scenario, even the initial part of the Raman probing pulse induces a process within the sample; as it propagates through the sample, it effectively generates and probes the process responsible for the background. As this pulse is in the spectral domain and constitutes a supercontinuum (quasi-white light), it effectively excites a wide spectral range of plasmonic resonances. The most likely process that could account for the generation of the background observed here involves plasmonic excitation followed by dissipative processes of the absorbed energy, such as the generation of hot electrons, their thermalisation to the crystal lattice, and ultimately, the dissipation of energy to the surroundings of the nanoantennas [36].

### 3.3 Spectral dependence of SE-FSRS signal

As illustrated by the analysis of band dependencies on parameters such as delay and pulse fluence, dispersive Raman bands associated with chemical compounds affixed to the nanoantennas consistently emerge against a broad spectral background. A broad background often appears in four-wave mixing experiments. Nonetheless, the origins and characteristics of this background in SE-FSRS have not been explored or elucidated, especially concerning their relationship with the distinctive dispersive profiles of the observed Raman bands.

**Figure 4:**
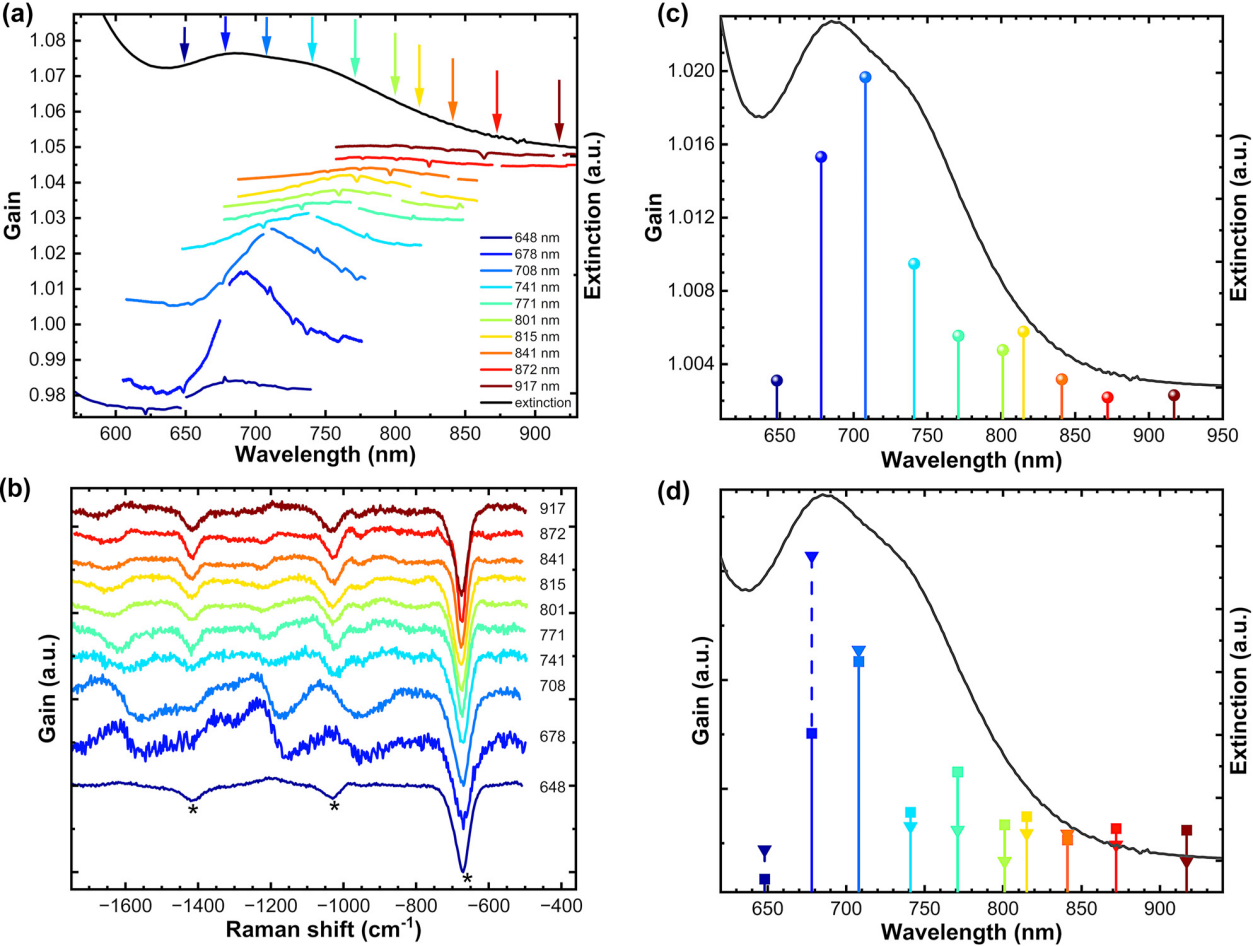
Influence of the spectral overlap between the excitation wavelength and the plasmon resonances of the silica/BPE/AuNPs colloidal solution on the SE-FSRS spectra. (a) Dependence of SE-FSRS spectra on excitation wavelength. (b) Background-corrected and normalised anti-Stokes branches of spectra in (a) adjusted relative to the intensity of the internal standard 670 cm^−1^ band of solvent DMSO. Stars indicate the positions of the DMSO bands. (c) Stationary extinction spectrum of the colloidal solution of silica/BPE/AuNPs (black line) and amplitude gain of the broadband background at its maximum. (d) Amplitudes of 1,201 cm^−1^ (squares) and 1,634 cm^−1^ (triangles) SE-FSRS bands of BPE as a function of the excitation wavelength.

A series of SE-FSRS spectra was recorded while continuously tuning the wavelength of the Raman pump pulses over a broad spectral range (Figures 4 and S7 in Supplementary Materials). The amplitude of the FSRS signal depends on the spatial and temporal overlap of the pump and probe pulses, which must be separately tuned for each excitation wavelength. Slight differences in the overlap of these pulses can affect the intensities of the collected spectra and increase the error in estimating the gain as a function of excitation wavelengths. Therefore, this experiment was also performed using a colloidal dispersion in DMSO in addition to aqueous colloids. Unlike water, DMSO exhibits easily measurable FSRS spectral bands. As the excitations are electronically non-resonant with DMSO, the amplitudes of its FSRS bands were used as an internal intensity standard.

Figure 4(a) shows that the intensity and shape of the broad background depended on the chosen excitation wavelength. The spectral position of the background maximum shifted with changes in the excitation wavelength, although its position did not maintain a constant spectral distance from the laser line (Figures 4(a) and S6 in Supplementary Materials). The spectral position of the maximum shifted toward the infrared region as the Raman pump wavelength was adjusted in this direction; however, the rate of this shift did not correlate consistently with the changes in wavelength. For low-wavelength excitations, the maximum appeared on the longer wavelength side, but for longer wavelengths, it appeared on the opposite side of the laser line. The amplitude of the gain at the peak of the positive part of the broad band appeared to be somewhat correlated with the spectral position of the Raman pump excitation relative to the extinction spectrum, as depicted in Figure 4(c). However, this correlation was relatively weak, with the most intense backgrounds occurring for excitations near the peak of the extinction spectrum. The amplitudes of the dispersive SE-FSRS bands of BPE behaved similarly when the Raman pump excitation wavelength was scanned (Figure 4(d)). However, the spectral dependence of the amplitudes for bands located at 1,201 and 1,634 cm^−1^ was not identical.

A close investigation of the shape of those bands also revealed that the dispersive shape changed with the change of the excitation wavelength. However, excitations at the 708 nm end below the dispersive shape showed a bottom-up slope when moving from lower to higher Raman shifts on the anti-Stokes branch of SE-FSRS spectra (Figure 4(b)), which changed to an up-down slope for excitations above 741 nm and maintained a very similar shape up to 911 nm excitation. Essentially, no significant change in shape or in the dispersive contribution to the band shape was observed upon changing the excitation wavelength over this spectral range. A closer examination of the dispersive bands, measured in smaller steps for a nanoparticle sample in water (Figure S6 in Supplementary Materials), revealed similar stability. This behaviour, a scan along the steep slope of the extinction band, suggests significant changes in the enhancement factor with excitation wavelength, yet only the amplitude of the dispersive bands’ changes, not their shape. This contradicts the predictions of Zong and Cheng [17], who argued through theoretical analysis that such changes should be pronounced, with dispersive shapes dominating at high enhancement factors and Lorentzian shapes at low ones. Furthermore, they also noted that the relative position of the band to the maximum of the extinction band should not influence the dispersive shape, which should only be dictated by the enhancement factor. Moreover, the observed reversal of the dispersive shape from up-down to down-up with increasing excitation wavelength (Figure 4(b)) complicates the interpretation of the origin of the dispersive shape. This change cannot be explained solely by different ratios of the real and imaginary components of Raman-dependent nonlinear susceptibility, as proposed by Zong and Cheng [17].

The above-mentioned analysis suggests that the complexity of the dependence on excitation may stem from the nature of the sample itself, which comprised a mixture of aggregates varying in size and shape, similar to previously reported SE-FSRS results [14], [15], [16], [29], [30], [31]. Due to the limitations in controlling the synthesis method, it was impossible to fully control the population of forming aggregates. As a result, the samples exhibited a specific distribution of nanoaggregate populations – from single nanospheres to their dimers, trimers, and beyond. Notably, aggregation appears necessary to form nanocavities where statistically distributed Raman reporter molecules (e.g. BPE) experience significantly higher plasmonic enhancements than those outside such cavities. The notably higher intensities observed in the generation of the broadband background and the dispersive SE-FSRS bands within a narrow range of excitation wavelengths suggest that aggregates with extinction peaks in this range exhibit the highest enhancement factor. The absence of SE-FSRS spectra observations from individual, separated nanoparticles implies that the enhancement occurring within nanocavities is crucial for SE-FSRS detection. The spectral behaviour of the background as a function of excitation reveals its correlation with plasmonic excitation. Due to differences in the extinction spectra of various aggregate types (e.g. dimers and trimers, Figure S3 in Supplementary Materials), populations of different aggregates are more efficiently excited by spectrally distinct excitations [37]. This complexity in the sample composition was manifested by the shifting position of the broadband background peak maximum (Figures 4(a) and S6 in Supplementary Materials). Measurements on single aggregates, or at least monodisperse samples, would simplify the complexity of the SE-FSRS response as a function of excitation and more strongly correlate it with plasmonic properties.

### 3.4 Origin of the broadband background

Gold nanobipyramids (AuBPs) were synthesised and encapsulated in a silica shell (Figure S2 in Supplementary Materials) to understand better how background band positions vary with excitation and to confirm the proposed source of the spectral shift in the broadband background observed in complex aggregate samples. Attempts were also made to synthesise analogous silica/BPE/AuBP samples to achieve a more correlated dependence of the SE-FSRS signal on the background; however, these did not produce a measurable SE-FSRS signal. However, nanoparticles coated with silica but not functionalised with BPE (silica/AuBP) exhibited an identical broad background, indicating that the source of this background was the nanoantenna itself.

**Figure 5:**
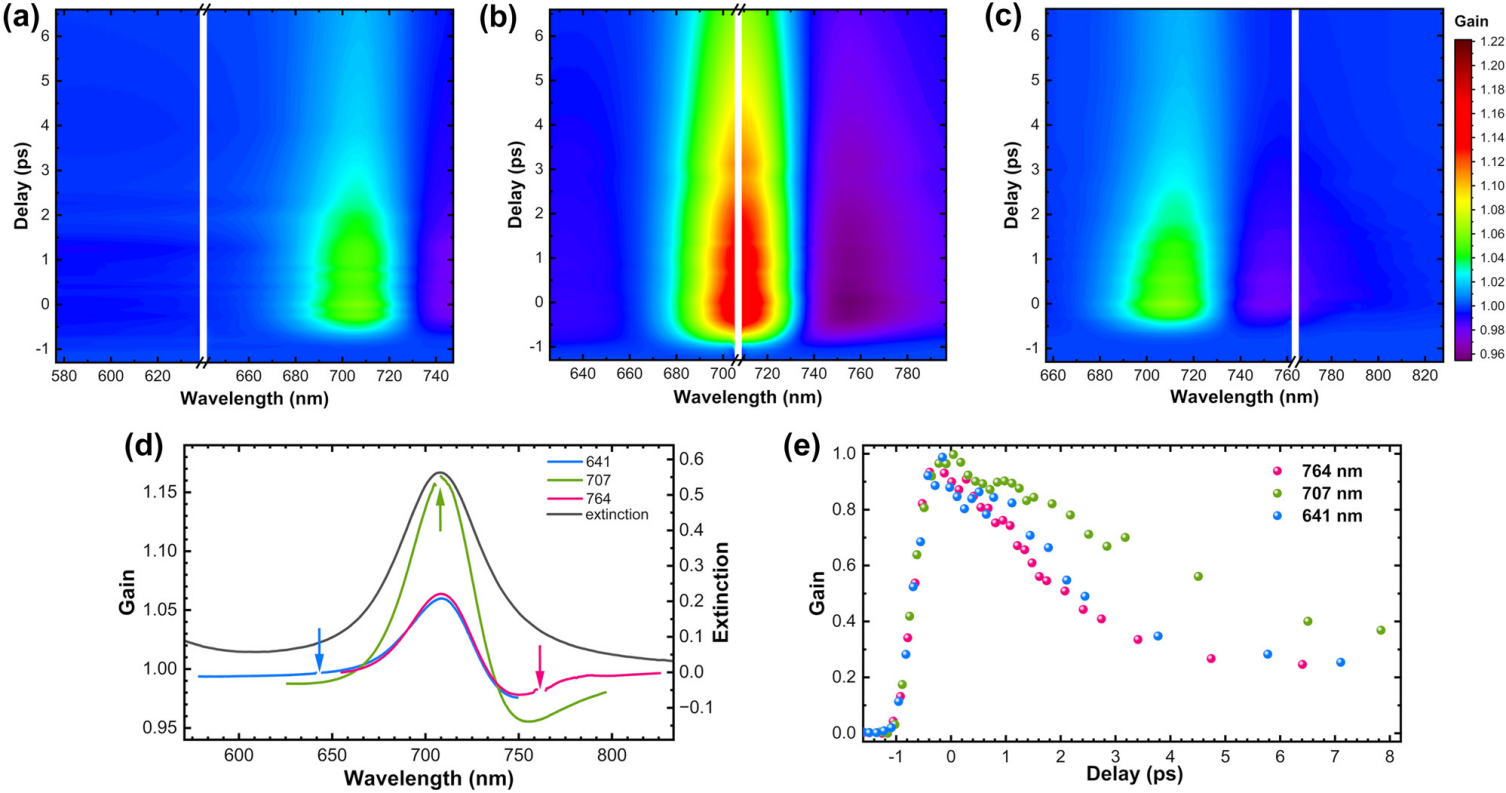
Temporal evolution of AuBPs colloid SE-FSRS spectra with pump beam excitation at wavelengths of (a) 641 nm, (b) 707 nm, and (c) 764 nm. (d) Extinction spectrum (black curve) and gain spectra excited at 641 nm (blue curve), 707 nm (green curve), and 764 nm (pink curve) with zero-time delay (d). Arrows indicate the spectral positions of the pump beam wavelengths. (e) Temporal evolution of the maximum intensity of the gained band for different excitation wavelengths.

The broadband signal observed in SE-FSRS experiments closely correlated with the extinction spectrum of the nanoantennas (Figure 5(c)), showing the same peak position but narrower half-widths. The shape of the band remained constant with changes in excitation wavelength, except for the total amplitude, which reached a maximum when excitation occurred at the extinction maximum. In addition to the band with positive gain, a broad band with negative gain also appears (Figure 5). Similar regions of negative gain were also observed in SE-FSRS spectra of samples with aggregated nanoparticles (Figures 2–4), where the spectral position of this negative gain band partially overlapped with the extinction band and shifted along with the positive gain band during the pump wavelength scan. In contrast, for non-aggregated AuBPs, the spectral positions of both the negative and positive gain parts of the spectrum remained fixed under different excitations, with only their amplitudes changing (Figure 5(d)). This phenomenon, characterized by the presence of spectral ranges with negative and positive gain, is most likely caused by changes in the sample’s extinction resulting from excitation by the Raman pulse. Given that the sequence of pump and probe pulses used in the FSRS measurements is analogous to those used in transient absorption (TA) spectroscopy, it can be inferred that the background in the SE-FSRS spectra primarily originated from changes in the TA of gold nanoparticles following their excitation by the Raman pump pulse. Based on studies of the TA spectra of gold nanoparticles, we can assume that the appearing in the SE-FSRS spectra broadband background was the result of plasmonic excitation, whose energy was dissipated into thermal form. Due to the different sizes and shapes of nanoparticles, as well as their material, the exact durations of the individual stages associated with energy redistribution after plasmonic excitation can vary. However, the general mechanism is now widely accepted and consists of several stages, which can partially overlap in time: dephasing (10 fs), electron-electron scattering (100 fs), electron-phonon scattering (1 ps), and coupling to the environment (10 ps) [36]. Each of the several stages of this energy dispersion can cause changes in the extinction of the nanoparticles, as reflected by the temporal evolution of such spectra. Due to the temporal resolution of the FSRS equipment, especially the pump pulse, which must last in the picosecond domain for the sake of Raman resolution, the broadband signal we registered is primarily related to the final phase of this energy dissipation process. In this phase, the heating of the nanoparticles and immediate environment of nanoparticles causes an alteration of the TA spectra.

Subsequent experiments were performed to investigate the temporal evolution of the broadband signal in relation to the delays between pulses (Figure 5(a)–(c)). The temporal resolution of the system was determined by the duration of the longer Raman pump pulse, which affected spectral resolution and was approximately 1 ps. The evolution of the spectrum extended over several tens of picoseconds (Figure S8 in Supplementary Materials), with the longest decay time occurring when the excitation spectrally overlapped with the extinction maximum.

Decay dynamics exhibited significant differences between aggregated spherical nanoparticles (Figure 3(d)) and non-aggregated gold bipyramids (Figure 5(e)). This variance appeared to be associated with the markedly smaller size of the spherical nanoparticles. Despite partial aggregation within the colloid, these nanoparticles primarily comprised very-small-volume monomers and dimers (Figures S2 and S3 in Supplementary Materials). As a result, the decay occurred much more rapidly, with its peak intensity diminishing at a rate similar to the duration of the pump pulse. Only a less intense tail of the decay signal persisted beyond 1 ps. In contrast, with AuBPs, the initial decay stage persisted for several picoseconds, and the overall process continued for at least several tens of femtoseconds. This outcome aligned with the findings of Hu et al. [38] and Chiang et al. [39], who noted significant variances in TA decay related to the shape and size of gold nanoparticles, and notably shorter decay times for smaller nanoparticles.

The registered, long-living temporal evolution of the SE-FSRS background resulted from heating and heat dissipation by the metallic nanoparticles. The heating induced by the pump pulse affected both the nanoparticles and their surrounding environment, leading to a local change in refractive index. As a result, the probing pulse beam underwent geometric alterations, which varied across different wavelengths. Consequently, the beam was focused differently on the detector, resulting in a measured spectral distribution reflecting gains or losses in intensity. The photothermal effect was also observed in SE-SRS relying on a nanoplasmonic waveguide [40], and it was proposed to be responsible for the dispersive shape of SE-SRS bands [17].

However, in the case of aggregates, we demonstrate that the temporal evolution of dispersive signals in the SE-FSRS spectra does not align in time, with the buildup and maximum of the broad background signal manifesting with a delay of several hundred femtoseconds (Figure 3(d)). No significant influence of the background intensity on the dispersive shape was observed, except for changes induced by the pump-probe time delay, which caused alterations similar to those observed in samples without nanoparticles (Figure 3(c)). The lack of such influence suggests that the background may have a source other than photothermal effects in these short sub-picosecond timescales. Furthermore, if the dispersion of band shapes was the result of photothermal effects, the lack of dependence of the degree of band dispersion on the intensity of this background in the temporal evolution of the spectra indicates that the background does not affect the shape of the SE-FSRS bands.

## 4 Conclusions

We reported the SE-FSRS of BPE adsorbed on spherical gold oligomers registered at a repetition rate 1 kHz. Our results demonstrated that the amplitude of the SE-FSRS signal increased as a function of fluence in a single Raman pump pulse. However, only the initial course of this increase was linear, and, after reaching its maximum value, a decrease in the amplitude of the signal was observed. Measurements of the SE-FSRS signal amplitude as a function of pump-probe time delay showed variability in both the SE-FSRS of BPE and broad background signal amplitudes. For the first time we have reported appearance of the broadband complex shape background signal assisting SE-FSRS spectra. To understand mechanism responsible for the appearance of this background, we used gold nanoparticles characterised by high monodispersity. FSRS measurements were conducted using AuBPs as a function of Raman pump pulse excitation wavelength and pump-probe time delay. FSRS spectra indicated that regardless of the Raman pulse wavelength, the background signal was fixed and overlapped with the extinction spectra of AuBPs. The evolution of the background amplitude as a function of pump-probe time delay proved its nature to be connected with the plasmonic excitation and following it thermal redistribution of the energy by plasmonic nanoparticles. The presented experimental results significantly enhance our understanding of surface-enhanced coherent Raman spectroscopy, especially in the context of theoretical description. In conclusion, our findings elucidate the spectral and temporal dimensions of SE-FSRS backgrounds, highlighting the role of nanostructure plasmonics and the critical influence of experimental conditions such as excitation wavelength and nanoparticle aggregation on the observed spectra.

The presented results enhance our understanding of the mechanisms that govern SE-FSRS and assist in refining experimental setups for more precise chemical and physical investigations; however, they also highlight discrepancies between experimental results and predictions based on existing theories. To better align with theoretical predictions, conducting experimental studies on monodispersive but aggregated samples (e.g. colloids comprising exclusively dimers of identical sizes) is essential. Such an approach would eliminate the averaging effects in the measured signal caused by a collection of highly diverse aggregates while simultaneously ensuring a robust signal. The findings from this research pave the way for the optimisation of SE-FSRS in practical applications, including rapid chemical sensing and single-molecule investigations.

## Supporting Materials

This file contains detailed information on the preparation of nanoparticles, their spectral and morphological characteristics, as well as additional SE-FSRS results.

## Supplementary Material

Supplementary Material Details

## Abbreviations

SE-FSRS: surface-enhanced femtosecond stimulated Raman scattering
FSRS: femtosecond stimulated Raman scattering
SERS: surface-enhanced Raman scattering
SM: Supplementary Materials
TA: transient absorption
BPE: *trans*-1,2-*bis*(4-pyridyl)ethene
