# Supplementary material for: Advancements in surface-enhanced femtosecond stimulated Raman spectroscopy: exploring factors influencing detectability and shapes of spectra
Source: Nanophotonics. 2024 Dec 6;14(1):1–12. doi: 10.1515/nanoph-2024-0272 (PMC11744450; doi:10.1515/nanoph-2024-0272)
Supplement: Supplementary file 1 — Supplementary Material Details [file j_nanoph-2024-0272_suppl_001.pdf]

Patryk Pyrcz and Sylwester Gawinkowski*

Advancements in Surface-Enhanced Femtosecond Stimulated Raman Spectroscopy: Exploring Factors Influencing Detectability and Shapes of Spectra

1 Preparation of optical nanoantennas

1.1 Materials

$HAuCl_4{\cdot}3H_2O$ (≥99.9 % trace metals basis), sodium citrate dihydrate (99 %), (3-aminopropyl)triethoxysilane (APTES) (97 %), sodium silicate solution (NaOH ≥ 10%, SiO_2 ≥ 27 %), tetraethyl orthosilicate (TEOS) (99.9 %), trans-1,2-bis(4-pyridyl)ethene (BPE) (97 %), hexadecyltrimethylammonium bromide (CTAB) (≥98.0 %) hexadecyltrimethylammonium chloride (CTAC) (25 wt. % in H_2O), silver nitrate ($AgNO_3$) (≥99.0 %), sodium borohydride ($NaBH_4$) (99 %), L-ascorbic acid (AA) (99 %), and dimethyl sulfoxide (DMSO) (99.9 %) were purchased from Sigma-Aldrich. The ammonia solution (28 %), hydrochloric acid (HCl) (35–38 %) and ethanol (EtOH) (HPLC, 99.9 %) were purchased from CHEMPUR. All reagents were used as purchased. Water used in the experiments was deionised (Elix, Millipore, >15 MΩ cm^{-1}). All glassware was thoroughly cleaned with an aqua regia solution and rinsed with water.

1.2 Synthesis protocols of gold nanoparticles

Spherical gold nanoparticles (AuNPs) have been obtained according to the seed growth procedure described in the literature [1, 2]. Seed nanoparticles were synthesised using the citrate reduction method developed by Turkevich *et al.* [3]. Briefly, a sodium citrate solution (340 mM, 0.5 mL) was added to a boiling $HAuCl_4$ (0.254 mM, 99.5 mL) with vigorous stirring. The solution was heated for 30 min at 90 °C. For the growth of the seed particles, stock solutions of $HAuCl_4$ (25 mM) and sodium citrate (60 mM) were prepared. 30 ml of seed was mixed with 20 ml of water, and 0.46 ml of 60 mM sodium citrate was added to the round-bottom flask. The mixture was heated to 90 °C for 5 min. After 5 min of heating, 0.23 ml of $HAuCl_4$ was added to the mixture twice with a time interval of 35 min. After adding two portions of $HAuCl_4$, 20 mL of water was added. In the second growth step, 0.956 mL of sodium citrate was added, and the solution was heated for 5 min. Then, two portions of $HAuCl_4$, each 0.478 mL, were added with a time interval of 35 min. In the addition of both portions, 27.2 mL of water was added. Repetition of the growth steps led to the formation of larger nanoparticles.

Gold bipyramids (AuBPs) were synthesised using the procedure described by Sánchez-Iglesias *et al.* [4]. However, instead of citric acid, we used sodium citrate dihydrate. The synthesis of AuBPs comprised two stages. A seed was obtained initially, while nanoparticles with the desired dimensions were produced in the subsequent growth stage. The seed was prepared by fast reduction of $HAuCl_4$ (10 mL, 0.25 mM) with freshly prepared ice-cold $NaBH_4$ (0.25 mL, 25 mM) in an aqueous CTAC solution (50 mM) in the presence of citric acid (5 mM) under vigorous stirring at room temperature. The mixture changed from light yellow to brown, indicating the formation of the seed. Subsequently, after approximately 2 min, the vial containing the seed was sealed and placed in an oil bath for 90 min at a temperature of 80 °C. The solution's colour was gradually changed during the heating process, transitioning from brown to red. After 90 min of heating, the vial with the seed was removed from the oil bath, allowed to cool, and subsequently stored at room temperature. At a temperature of 30 °C, a growth solution was prepared, composed of CTAB (50 mL, 100 mM), $HAuCl_4$ (2.5 mL, 10 mM), $AgNO_3$ (0.5 mL, 10 mM), HCl (1 mL, 1 M), and AA (0.4 mL, 100 mM). The seed solution, with a volume of 1.25 mL, was added to the growth solution under vigorous stirring. The mixture was allowed to stand for at least 2 h with gentle stirring.

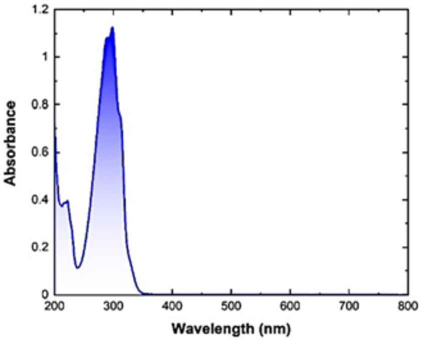

Fig. S1: The absorption spectrum of BPE in water (10^{-5} M).

*Corresponding author: Sylwester Gawinkowski, Institute of Physical Chemistry, Polish Academy of Sciences, 01-224 Warsaw, Kasprzaka 44/52, Poland; sgawinkowski@ichf.edu.pl; ORCID: 0000-0001-5315-6197
First author: Patryk Pyrcz, Institute of Physical Chemistry, Polish Academy of Sciences, 01-224 Warsaw, Kasprzaka 44/52, Poland; ppyrcz@ichf.edu.pl; ORCID: 0000-0003-4890-7384

1.3 Optical nanoantenna characterisation

Silica-coated gold optical nanoantennas were characterised using 10 kV scanning electron microscope (SEM) (FEI Nova NanoSEM 450) and UV/VIS/NIR spectrophotometer (Shimadzu 3100). Typical SEM images of the two types of samples are shown in Figure S2. The size of the AuNPs was estimated to be approximately 50 nm. The thickness of the silica coating was approximately 60 ± 11 nm. The size of AuBPs was estimated to be 49 ± 6 nm (length) and 18 ± 2 nm (width). The thickness of the silica shell was about 70 nm. The distribution of individual fractions was around 58.75 % monomers, 25 % dimers, 8.5 % trimers, 3.35 % tetramers, 1.5 % pentamers, and 2.9 % larger oligomers. In the case of silica/AuBPs, most nanoparticles were single-core.

The extinction spectra for all types of optical nanoantennas were measured from 400 to 1100 nm. Table S1 presents the spectral characteristics of optical nanoantennas, which include the location of surface plasmon resonances and optical density during SERS and SE-FSRS. The single AuNP mode corresponds to the plasmon resonance of the single-core nanoparticles, whereas the extinction peak for oligomers is shifted towards longer wavelengths. The extinction peak for oligomers is much broader than for monomers due to the plasmon resonances of the multiple-core nanoparticles.

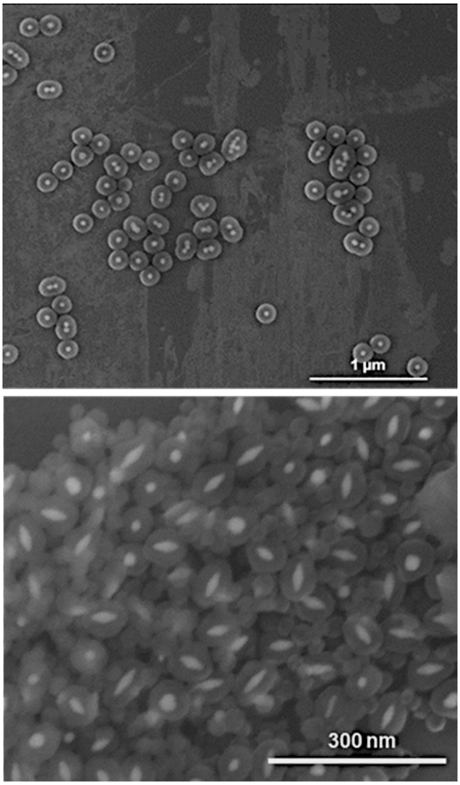

Fig. S2: SEM images showing silica/BPE/AuNPs (upper panel) and silica/AuBPs (lower panel).

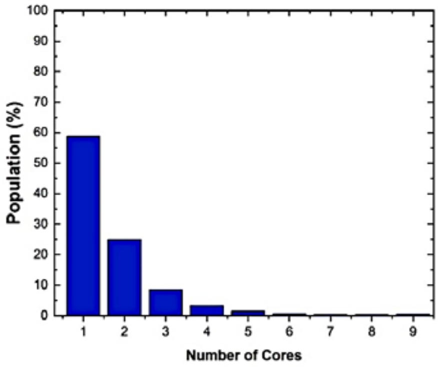

Fig. S3: Histogram of the population percentage as a function of the number of silica/BPE/AuNPs cores.

Nanoparticle	1st SPR	2nd SPR	OD at 1st SPR
silica/BPE/AuNPs in H_2O	537 nm	640 – 850 nm	0.80
silica/BPE/AuNPs in DMSO	538 nm	630 – 870 nm	0.80
silica/AuBPs in H_2O	526 nm	625 – 790 nm	0.56

Tab. S1: Spectral characteristics of optical nanoantennas determined based on extinction measurements.

2 Comparison of experimental spectra (normal Raman (NR), SERS, FSRS and SE-FSRS) of BPE with simulated spectrum (DFT)

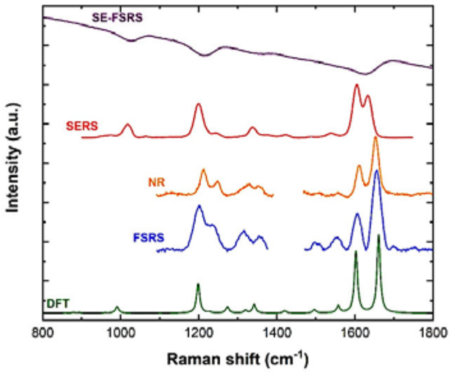

Fig. S4: Spectra of 50 mM BPE in DMSO obtained using normal Raman scattering (excitation at 661 nm, orange) and FSRS (excitation at 665 nm, blue), with solvent bands removed from both. SERS (excited at 671 nm, red) and SE-FSRS (excited at 665 nm, violet) spectra registered on silica/BPE/AuNPs. The B3LYP/6-31G(d,p) simulated spectrum for BPE in vacuum (green).

3 FSRS data examining time-delay dependence between the pump and probe pulses for BPE in DMSO

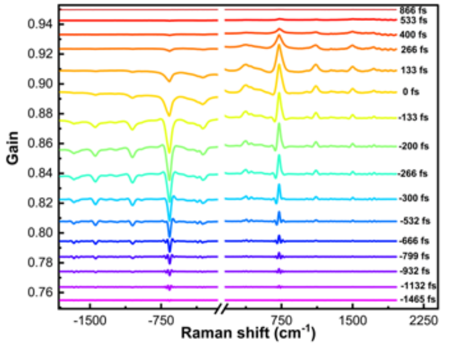

Fig. S5: FSRS spectra of a 50 mM BPE solution in DMSO, recorded with varying time-delay between the Raman pump and probe pulses, using a Raman pump excitation wavelength of 665 nm.

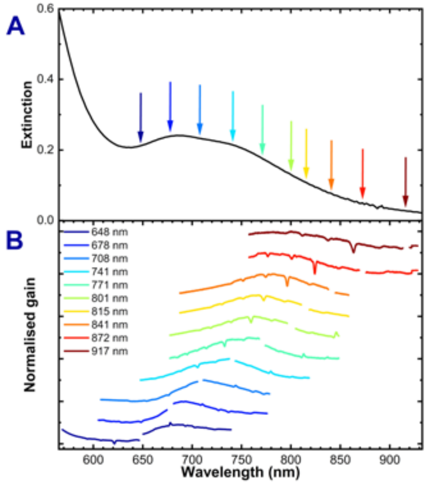

Fig. S6: Extinction spectrum of the silica/BPE/AuNPs colloid (A) and SE-FSRS spectra, measured using different Raman pump laser wavelengths and normalised to the maximum intensity of the broad band (B).

4 Additional SE-FSRS data as a function of excitation wavelength for silica-coated BPE-embedded spherical gold oligomers (silica/BPE/AuNPs)

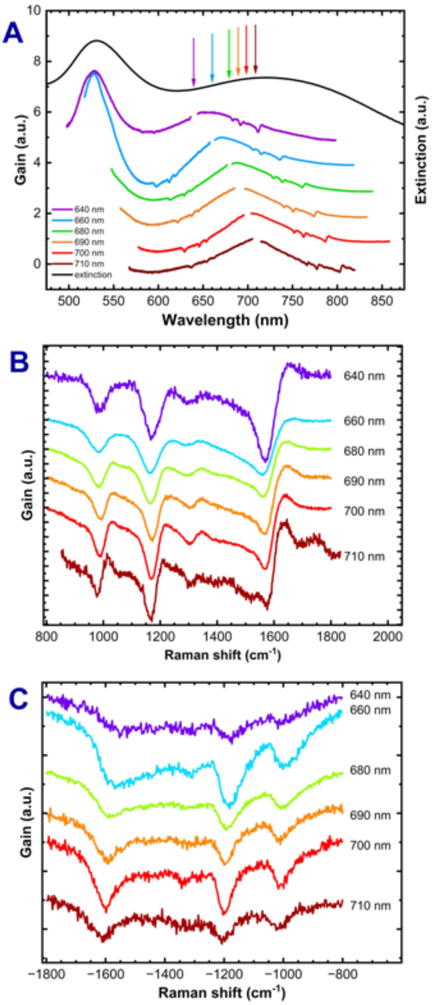

Fig. S7: Extinction spectrum (black line) and SE-FSRS spectra (coloured lines) with different excitation wavelengths recorded on silica/BPE/AuNP colloid with PRs for oligomers in the range 650–900 nm (A). Contributions from the Raman pump laser line have been removed from the spectra. The intensities of the SE-FSRS spectra have been normalised. The background-corrected and normalised Stokes (B) and anti-Stokes (C) branches of the SE-FSRS spectra.

5 Additional SE-FSRS data examining time-delay dependence between the Raman pump and probe pulses for silica-coated gold bipyramids (silica/AuBPs)

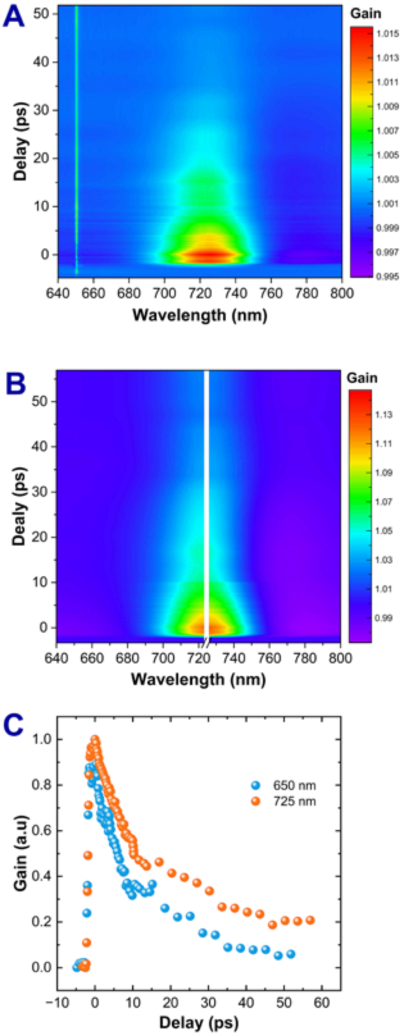

Fig. S8: Evolution of SE-FSRS spectra with varying time-delay between the Raman pump and probe pulses for silica/AuBPs, measured at Raman pump excitation wavelengths of 650 nm (A) and 725 nm (B). Normalised broadband gain decays at 720 nm (C). Spectra were recorded using a pump pulse energy of 5 nJ and an acquisition time of 10 seconds.

The broadband signal gain measurements were performed as functions of a time delay up to 50 ps to accurately describe the background and present its duration. Measurements of the background amplitude as a function of time delay were performed for silica/AuBPs (55 ± 4) nm × (21 ± 1) nm with an SPR band in the 650–790 nm range. The nanoparticles used were larger than those used in the amplitude versus time delay measurements in the range of up to 8 ps (manuscript). Background amplitude measurements were performed by comparing two distant excitations – 650 nm and 725 nm. The results are presented as a 2D map in Figure S8 (A and B). A background decay profile was plotted from these measurements (Figure S8C).

6 Calculation of electron temperature change by using a two-temperature model

The two-temperature model (TTM), which is effective in interpreting transient absorption results [5-7], was used to analyse our experimental data. This model was employed to estimate the initial electron temperature. TTM describes the coupling between the hot electrons and the phonon modes of the nanoparticle [5, 8]. However, it is essential to emphasise that the following analysis is approximate due to the model's limitations regarding temperature [9]. Specifically, the temperatures we obtained exceed the range for which the model is applicable.

Finite-difference time-domain (FDTD) simulations were performed using the commercial Ansys Lumerical software to compute the cross-sections for absorption and scattering. The AuBP was modelled as two back-to-back stacked 5-sided frustums of a pyramid. The shape of the silica shell was designed as an ellipsoid, with sizes set according to the average diameter and length measured from the SEM image. During the simulations, the electromagnetic pulse was modelled as circularly polarised light (TFSF). A mesh size of 0.5 nm was employed for calculating absorption and scattering cross-sections. The refractive index of the surrounding medium was set to that of water (1.33), and the refractive index of SiO_2 was set to 1.4. The dielectric function of Au was obtained by fitting the measured data of Johnson and Christy. The results of the simulation are presented in Table S2.

In summary, in TTM, the rate of energy exchange between electrons and phonons is described by coupled differential equations [5, 10]:

$$C_e(T_e)\frac{dT_e}{dt} = -g(T_e - T_l)$$

$$C_l\frac{dT_l}{dt} = g(T_e - T_l)$$

where T_e and T_l are the electronic and lattice temperatures, C_l is the lattice heat capacity,

$$C_e(T_e) = \gamma T_e$$

is the temperature dependent electronic heat capacity, and g is the electron−phonon coupling constant. The temperature dependence of C_e means that the time scale for electron−phonon coupling depends on the initial electronic temperature.

The change in electron temperature (ΔT_e) was calculated as a function of the Raman pump wavelength using the experimental extinction spectra and simulated optical cross-sections (abbreviations and values for parameters used in these calculations are provided in Table S2):

$$\Delta T_e = \sqrt{{T_0}^2 + \frac{2 \times \%abs \times E}{N \times V \times \gamma}} - T_0$$

For the data in Figure 4 (A-C) of the main text, the maximum electron temperature changes calculated for various excitations of the Raman pump pulse are as follows: 4492 K excited at 641 nm, 4195 K excited at 764 nm, and 7733 K excited at 707 nm. On the basis of these calculated electron temperature values, we determined the electron heat capacity, as the electronic heat capacity depends on the electron distribution temperature. The results are: $3.0 \cdot 10^5$ J K^{-1} m^{-3} excited at 641 nm, $2.8 \cdot 10^5$ J K^{-1} m^{-3} excited at 764 nm, and $5.2 \cdot 10^5$ J K^{-1} m^{-3} excited at 707 nm. It should be emphasised that these values were determined for the initial electron temperature immediately after pump laser excitation. Over time, the colloid tends to reach equilibrium with its surroundings, leading to energy transfer to the metal lattice, thus increasing its temperature [11].

The decay profiles presented in Figure 4E indicate an exponential decrease in the background signal amplitude, which corresponds to a temperature decay due to energy transfer to the metal lattice. Simulations suggest that the metal lattice temperature is approximately 6–7 times lower than the initial electron temperature [12]. When comparing the specific electronic heat capacity with the lattice heat capacity (for bulk gold = $2.4 \cdot 10^6$ J K^{-1} m^{-3}) [12], we observe a similar value, suggesting potential expansion of the nanoparticle without causing its destruction. Frontiera *et al.* observed a decrease in the extinction of optical nanoantennas in the NIR plasmon resonance region after several weeks of exposure. However, they did not observe any abnormally fused or melted nanoparticles via transmission electron microscopy (TEM), but only lighter contrast rings around the silica shells. This observation is consistent with our predictions supported by the TTM model approximation.

Tab. S2: Data used to calculate the change in electron temperature using the TTM model. The cross-section values were obtained from FDTD simulations.

Parameter	Meaning	Value [Unit]
T_0	ambient temperature	293.15 K
N	the number of AuBPs illuminated by the pump beam	$1.78 \cdot 10^5$
V	nanoparticle volume	$2.22 \cdot 10^{-24}$ m^3
E	the average energy of the pump beam	$5 \cdot 10^{-9}$ J
Υ	electron heat capacity constant	66 J m^{-3} K^{-2}
σ_{abs}	absorption cross-section	$5.78 \cdot 10^{-15}$ m^2
σ_{scat}	scattering cross-section	$4.29 \cdot 10^{-16}$ m^2
σ_{ext}	extinction cross-section	$6.21 \cdot 10^{-15}$ m^2

References

[1] J. H. Yoon, J. Lim, and S. Yoon, "Controlled assembly and plasmonic properties of asymmetric core-satellite nanoassemblies," *ACS Nano*, vol. 6, no. 8, pp. 7199–7208, 2012, https://doi.org/10.1021/nn302264f.

[2] N. G. Bastús, J. Comenge, and V. Puntes, "Kinetically controlled seeded growth synthesis of citrate-stabilised gold nanoparticles of up to 200 nm: Size focusing versus Ostwald ripening," *Langmuir*, vol. 27, no. 17, pp. 11098–11105, 2011, https://doi.org/10.1021/la201938u.

[3] J. Turkevich, P. C. Stevenson, and J. Hillier, "A study of the nucleation and growth processes in the synthesis of colloidal gold," *Discuss. Faraday Soc.*, vol. 11, no. c, pp. 55–75, 1951, https://doi.org/10.1039/DF9511100055.

[4] A. Sánchez-Iglesias, N. Winckelmans, T. Altantzis, S. Bals, M. Grzelczak, and L. M. Liz-Marzán, "High-yield seeded growth of monodisperse pentatwinned gold nanoparticles through thermally induced seed twinning," *J. Am. Chem. Soc.*, vol. 139, no. 1, pp. 107–110, 2017, https://doi.org/10.1021/jacs.6b12143.

[5] G. V. Hartland, "Optical studies of dynamics in noble metal nanostructures," *Chem. Rev.*, vol. 111, no. 6, pp. 3858–3887, 2011, https://doi.org/10.1021/cr1002547.

[6] C. K. Sun, F. Vallée, L. H. Acioli, E. P. Ippen, and J. G. Fujimoto, "Femtosecond-tunable measurement of electron thermalisation in gold," *Phys. Rev. B*, vol. 50, no. 20, pp. 15337–15348, 1994, https://doi.org/10.1103/PhysRevB.50.15337.

[7] J. Hohlfeld, S. S. Wellershoff, J. Güdde, U. Conrad, V. Jähnke, and E. Matthias, "Electron and lattice dynamics following optical excitation of metals," *Chem. Phys.*, vol. 251, no. 1–3, pp. 237–258, 2000, https://doi.org/10.1016/S0301-0104(99)00330-4.

[8] J. Hodak, I. Martini, and G. V. Hartland, "Ultrafast study of electron-phonon coupling in colloidal gold particles," *Chem. Phys. Lett.*, vol. 284, no. 1–2, pp. 135–141, 1998, https://doi.org/10.1016/S0009-2614(97)01369-9.

[9] A. M. Brown, R. Sundararaman, P. Narang, W. A. Goddard, and H. A. Atwater, "Ab initio phonon coupling and optical response of hot electrons in plasmonic metals," *Phys. Rev. B*, vol. 94, no. 7, pp. 1–10, 2016, https://doi.org/10.1103/PhysRevB.94.075120.

[10] W. Y. Chiang et al., "Electron-Phonon Relaxation Dynamics of Hot Electrons in Gold Nanoparticles Are Independent of Excitation Pathway," *J. Phys. Chem. C*, vol. 127, no. 43, pp. 21176–21185, 2023, https://doi.org/10.1021/acs.jpcc.3c04680.

[11] C. Voisin, N. Del Fatti, D. Christofilos, and F. Vallee, "Ultrafast electron dynamics and optical nonlinearities in metal nanoparticles," *J. Phys. Chem. B*, vol. 105, no. 12, pp. 2264–2280, 2001, https://doi.org/10.1021/jp0038153.

[12] J. H. Hodak, I. Martini, and G. V. Hartland, "Spectroscopy and dynamics of nanometer-sized noble metal particles," *J. Phys. Chem. B*, vol. 102, no. 36, pp. 6958–6967, 1998, https://doi.org/10.1021/jp9809787.

[13] R. R. Frontiera, A. I. Henry, N. L. Gruenke, and R. P. Van Duyne, "Surface-enhanced femtosecond stimulated Raman spectroscopy," *J. Phys. Chem. Lett.*, vol. 2, no. 10, pp. 1199–1203, 2011, https://doi.org/10.1021/jz200498z.
